# Identification of long non-coding RNAs that stimulate cell survival in bladder cancer

**DOI:** 10.18632/oncotarget.16284

**Published:** 2017-03-16

**Authors:** Aleksandra M. Dudek, Sabrina J. Boer, Nanda Boon, J. Alfred Witjes, Lambertus A.L.M. Kiemeney, Gerald W. Verhaegh

**Affiliations:** ^1^ Department of Urology, Radboud University Medical Center, Radboud Institute for Molecular Life Sciences, Nijmegen, The Netherlands; ^2^ Department for Health Evidence, Radboud University Medical Center, Radboud Institute for Health Sciences, Nijmegen, The Netherlands

**Keywords:** long non-coding RNA, bladder cancer, MiTranscriptome, lncRNA, biomarker

## Abstract

For many years, research on the biology underlying bladder cancer focused on protein-coding genes which cover only about 3% of the human genome. Recently, it was discovered that a large part of the human genome is actively transcribed as long non-coding RNAs (lncRNAs). LncRNAs are master regulators of gene expression and several lncRNAs were shown to play a role in bladder cancer development and progression. Here, we analyzed lncRNA expression in muscle-invasive bladder cancer (MIBC) using the MiTranscriptome database of cancer lncRNA expression profiles, and we studied their function in bladder cancer-derived tumor cells. Analysis of the MiTranscriptome lncRNA expression data revealed four MIBC subgroups, which partially overlapped with the four mRNA clusters identified by The Cancer Genome Atlas consortium. Up-regulation of three lncRNAs *CAT266, CAT1297*, and *CAT1647* in bladder cancer, in comparison to normal urothelium, was confirmed in an independent series of normal, non-muscle invasive (NMIBC) and MIBC tissue samples. Furthermore, expression levels of *CAT1297* were found to be correlated with disease-free and overall survival in MIBC. Knockdown of *CAT266, CAT1297*, and *CAT1647* decreased cell viability and colony formation, due to the induction of apoptosis. In conclusion, our data show that lncRNAs expression is de-regulated in MIBC and three aberrantly expressed transcripts regulate proliferation and apoptosis. Our data indicate that lncRNAs play an important role in MIBC development and progression and are a treasure chest for the discovery of new biomarkers.

## INTRODUCTION

Bladder cancer is the most common malignancy of the urinary tract worldwide. Accurate diagnostic and prognostic biomarkers and effective treatments are lacking and represent major challenges in urological oncology [1]. Bladder cancer is a heterogeneous disease. Approximately 75 to 85% of patients are diagnosed with non-muscle invasive bladder cancer (NMIBC), which is characterized by a high recurrence rate (ranging from 30 to 80% in five years). Due to extensive follow-up and treatment of NMIBC the cost per patient is highest of all cancers [2]. The remaining group of patients (~15 to 25%), present with muscle-invasive bladder cancer (MIBC) that often progresses to metastatic disease. 5-year survival after a diagnosis of MIBC is only approximately 50% [3].

For many years, research on the biology underlying bladder cancer focused on protein-coding genes [1]. Protein-coding sequences cover only about 3% of the human genome [4]. However, a large part of the human genome is actively transcribed and most of the transcriptional output is represented by non-coding RNAs (ncRNAs). One class of ncRNAs are the long non-coding RNAs (lncRNAs), which are transcripts of more than 200 nucleotides lacking protein-coding potential [4, 5]. Recently, a systematic analysis of all publicly available next generation sequencing data revealed that over 60% of genes expressed in normal and tumor tissues are in fact lncRNAs and that their expression is often lineage and even cancer-specific [5].

LncRNAs are key regulators of gene expression, acting both in the nucleus and in the cytoplasm. Nuclear lncRNAs influence gene expression in *cis* and *trans* by the recruitment of chromatin modifiers and interaction with transcription factors. LncRNAs located in the cytoplasm affect gene expression post-transcriptionally by modulating mRNA translation, stability or decay. Furthermore, lncRNAs may act as microRNA sponges, modulating the cellular levels of microRNAs and, hence, expression of their target genes [6].

Cancer development is associated with deregulation in gene expression, and aberrant lncRNA expression was shown to be implicated in the development of many different cancer types [7]. Also in bladder cancer several oncogenic and tumor suppressive lncRNAs have been identified, such as *H19*, *MALAT1, MEG3, SNHG16, TUG1* and *UCA1* [8]. LncRNA expression levels often correlate with prognosis and metastasis formation [9, 10] or occurrence of therapy resistance [11]. In bladder cancer, the up-regulation of *UCA1* was found to induce EMT, tumor cell migration and invasion [12] and it was shown to contribute to cisplatin and gemcitabine resistance [13]. Because of their significant role in cancer development lncRNAs might be potential targets for development of new therapies [14].

In the present study, we evaluated expression levels of lncRNAs in MIBC using the publicly available MiTranscriptome cancer lncRNA expression data [5]. Aberrant expression of three up-regulated transcripts (*CAT266*, *CAT1297* and *CAT1647*) was confirmed in an independent series of normal urothelium, NMIBC and MIBC tissue samples. Antisense oligonucleotides were used to knockdown lncRNA expression in bladder cancer cell lines. We found that reduced expression of *CAT266*, *CAT1297* and *CAT1647* decreased cell viability and induced apoptosis. Our data suggest that lncRNAs play an important role in bladder cancer development and progression, indicating that MiTranscriptome lncRNAs expression data can be use as a platform for the discovery of new biomarkers and therapeutic targets for bladder cancer.

## RESULTS

### Characterization of long non-coding RNAs expression in MIBC

**Figure 1:**
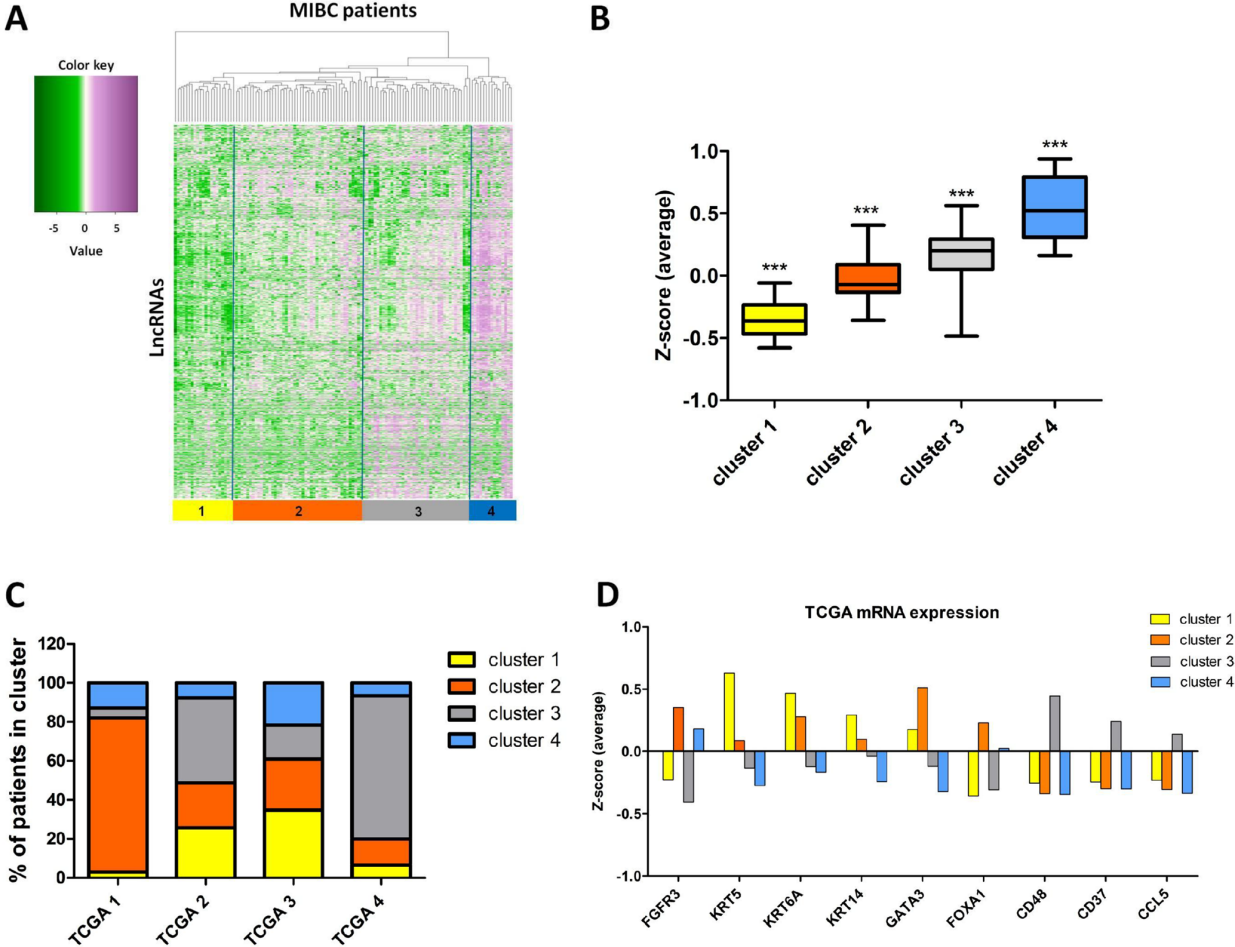
Characterization of lncRNAs expression in MIBC (**A**) Hierarchical clustering analysis of lncRNA expression in 122 MIBC tissue samples identified 4 MIBC lncRNA clusters. FPKM data are log transformed and standardized by Z-score transformation. Green, negative Z-score; purple, positive Z-score. (**B**) The average expression levels (Z-score) of lncRNAs in the 4 identified clusters. Each cluster is characterized by significantly different average Z-scores (*p <* 0.001). (**C**) Overlap between 4 MIBC lncRNA clusters (cluster 1-4) and TCGA mRNA clusters (TCGA 1-4) [23]. (**D**) Expression levels of mRNAs defining TCGA clusters. The expression levels are shown as average Z-scores in each of the lncRNA clusters. The bars represent mean.

To determine the expression pattern of long non-coding RNAs in MIBC, we analyzed expression of 12,382 transcripts identified by Iyer *et al*. [5], who reanalyzed TCGA RNA-seq data. Heterogeneity of MIBC patients was revealed by hierarchical clustering of 2,504 de-regulated lncRNAs into 4 distinct lncRNA expression clusters (cluster #1-4, Figure 1A). Cluster 1 samples were characterized by the lowest overall expression of lncRNAs, cluster 2 and 3 samples showed intermediate levels of lncRNAs, whilst cluster 4 was characterized by overall the highest level of lncRNA expression (Figure 1B). Furthermore, lncRNA cluster 1 samples showed an overlap with TCGA cluster 2 and 3 (25.6 and 34.8%, respectively) (Figure 1C), and showed higher *KRT5*, *KRT6A* and *KRT14* mRNA expression, characteristic for basal/squamous tumors (TCGA cluster 3) (Figure 1D). LncRNA cluster 2 was over-represented in papillary-like TCGA cluster 1 (79.2% of samples, Figure 1C), marked by high expression of *FGFR3*, but also high expression of luminal differentiation markers *GATA3* and *FOXA1* (Figure 1D). LncRNA cluster 3 overlapped with TCGA cluster 2 and 4 (43.6 and 73.3% of samples) (Figure 1C) and showed high expression of immune-related genes (*CD48*, *CD37* and *CCL5*) (Figure 1D). LncRNA cluster 4 was found to be overrepresented in TCGA cluster 1 and 3 (12.8 and 21.7% of samples) (Figure 1C), marked by higher *FGFR3* expression, but in contrast to cluster 2 lower expression of *GATA3* and *FOXA1* (Figure 1D).

### *CAT266, CAT1297* and *CAT1647* are up-regulated in bladder cancer

**Figure 2:**
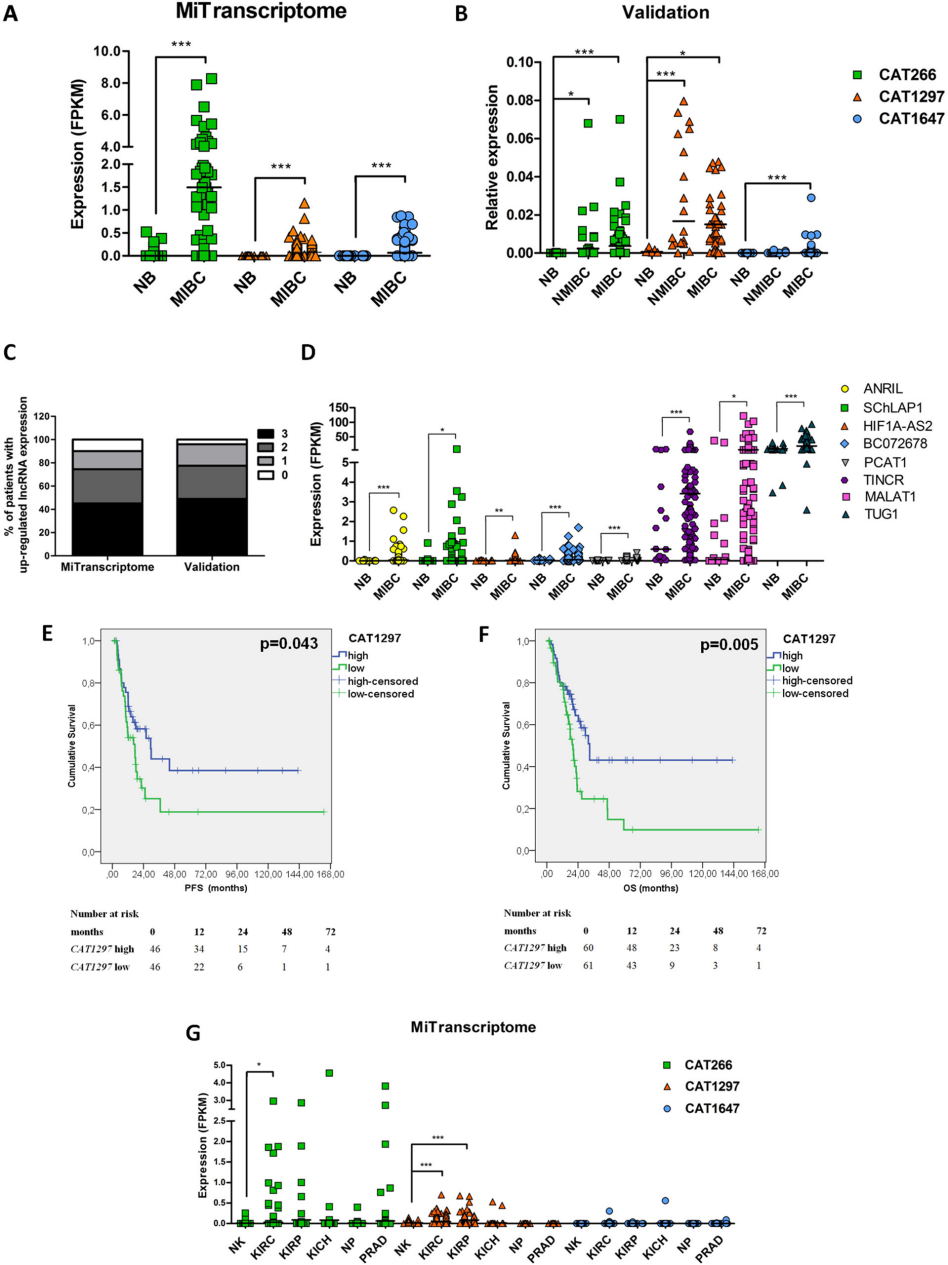
Expression of *CAT266, CAT1297* and *CAT1647* in normal urothelium (NB), non-muscle-invasive (NMIBC) and muscle-invasive bladder cancer (MIBC) in (**A**) MiTranscriptome discovery set and (**B**) in the Nijmegen validation set. Expression data were analyzed using unpaired *t-test* (*p < 0.05; **p < 0.01; ***p < 0.001, line represent the median). (**C**) Percentage of MIBC patients with 3, 2, 1, 0 up-regulated lncRNA in the MiTranscriptome discovery set and in the validation set in comparison to median expression levels of each lncRNA in normal urothelium. (**D**) Expression levels of lncRNAs described as up-regulated in bladder cancer in literature, in the MiTranscriptome cohort of MIBC. Expression data were analyzed using unpaired *t-test* (*p < 0.05; **p < 0.01; ***p < 0.001, line represent the median). (**E**) Kaplan-Meier analysis, using data from the MiTranscriptome cohort, showing correlation of *CAT1297* expression levels (high *n* = 46; low *n* = 46) with progression-free survival (PFS) and (**F**) overall survival (OS; high *n* = 60; low *n* = 61). (**G**) The expression levels of *CAT266*, *CAT1297* and *CAT1647* in other urological tissue types: normal kidney (NK, *n* = 119), kidney renal clear cell carcinoma (KIRC, *n* = 463), kidney renal papillary cell carcinoma (KIRP, *n* = 77), kidney chromophobe renal cell carcinoma (KICH, *n* = 64), normal prostate (NP, *n* = 43) and prostate adenocarcinoma (PRAD, *n* = 174) in the MiTranscriptome dataset. Expression data were analyzed using unpaired *t-test* (*p < 0.05; **p < 0.01; ***p < 0.001, line represent the median).

Of the 12,382 lncRNAs that are expressed in MIBC, 892 transcripts were found to be significantly down-regulated in MIBC, whereas 724 transcripts were up-regulated at least 2-fold (*p <* 0.05 unadjusted for multiple comparisons). We focused on up-regulated transcripts, as these are more likely to become implemented as a biomarker, and selected three aberrantly expressed transcripts (Figure 2A, Supplementary Table 3), namely *CAT1297* and *CAT1647* (the highest tumor/normal ratio based on average expression levels) and *CAT266* (the highest tumor/normal ratio based on median expression levels). The up-regulation of these three lncRNAs in MIBC in comparison to normal urothelium was confirmed in an independent set of muscle-invasive bladder cancer and normal urothelium tissue samples, with at least 17-fold increase in expression levels (*p <* 0.001, *p <* 0.05, *p <* 0.001, resp.) (Figure 2B). *CAT1647* was found to be MIBC-specific, whereas *CAT266* and *CAT1297* were also found to be up-regulated in NMIBC (*p <* 0.05 and *p <* 0.001, resp.) (Figure 2B). In the majority of MIBC tumors (90.16% in the MiTranscriptome cohort, and 95.92% in the Nijmegen validation cohort), at least one of the selected lncRNAs (*CAT266, CAT1297, CAT1647*) was up-regulated relative to the median expression levels of each lncRNA in normal urothelium (Figure 2C). Interestingly, up-regulation of *CAT266* and *CAT1297* was not associated with TCGA-identified MIBC subtypes, whereas expression of *CAT1647* was found to be higher in the basal TCGA cluster 3 (Supplementary Figure 1).

Finally, we evaluated the expression of bladder cancer-associated lncRNAs that have been described in literature. Elevated expression of *ANRIL* [15], *SChLAP1* [16], *HIF1A-AS2* [17], *BC072678* [18], *PCAT1* [19], *TINCR* [20], *MALAT1* [21], *TUG1* [22] in MIBC was confirmed in the MiTranscriptome analysis, however with much lower fold changes than was found for *CAT266*, *CAT1297* and *CAT1647* (Figure 2D, Supplementary Table 4).

### Association of lncRNA expression and tumor progression in the MiTranscriptome cohort

Expression levels of *CAT1647*, *CAT1297*, and *CAT266* were correlated with clinico-pathological parameters (Supplementary Table 5). Expression levels of *CAT1647* were found to be significantly higher in high grade (*p* = 0.027) and pT2 tumors (*p* = 0.015), whereas expression of *CAT1297* was significantly associated with male gender (*p* = 0.008). Furthermore, expression levels of *CAT1297* significantly correlated with progression-free survival (*p <* 0.05) (Figure 2E) and overall survival (*p <* 0.01) (Figure 2F). Interestingly, despite the fact that *CAT1297* is associated with MIBC development, patients expressing *CAT1297* at higher levels showed significantly longer PFS and OS. No correlation of *CAT1647* and *CAT266* expression with PFS or OS was found. In multivariate analysis, *CAT1297* expression remained an independent prognostic factor of MIBC overall survival (Supplementary Table 6).

### Tissue specific expression of MIBC-associated lncRNAs

The MIBC-associated lncRNAs could serve as potential biomarkers to diagnose and monitor bladder cancer, using *e.g*. urine as an analyte. We evaluated their expression in other urological tissue types and tumors (also available in the TCGA project). Expression of *CAT1297* was found to be significantly up-regulated in clear cell carcinoma and papillary cell carcinoma of the kidney compared to normal kidney (*p <* 0.001), and *CAT266* was found to be up-regulated only in renal clear cell carcinoma (*p <* 0.05). No de-regulated expression of *CAT1647* in different subtypes of kidney cancer was found. None of three MIBC-associated lncRNAs were found to be significantly deregulated in prostate cancer in comparison to normal prostate tissue. Most importantly, all three transcripts (*CAT266, CAT1297* and *CAT1647*) are expressed at very low levels in normal kidney and normal prostate tissue (Figure 2G).

### Functional effects of *CAT266, CAT1297* and *CAT1647* knockdown

**Figure 3:**
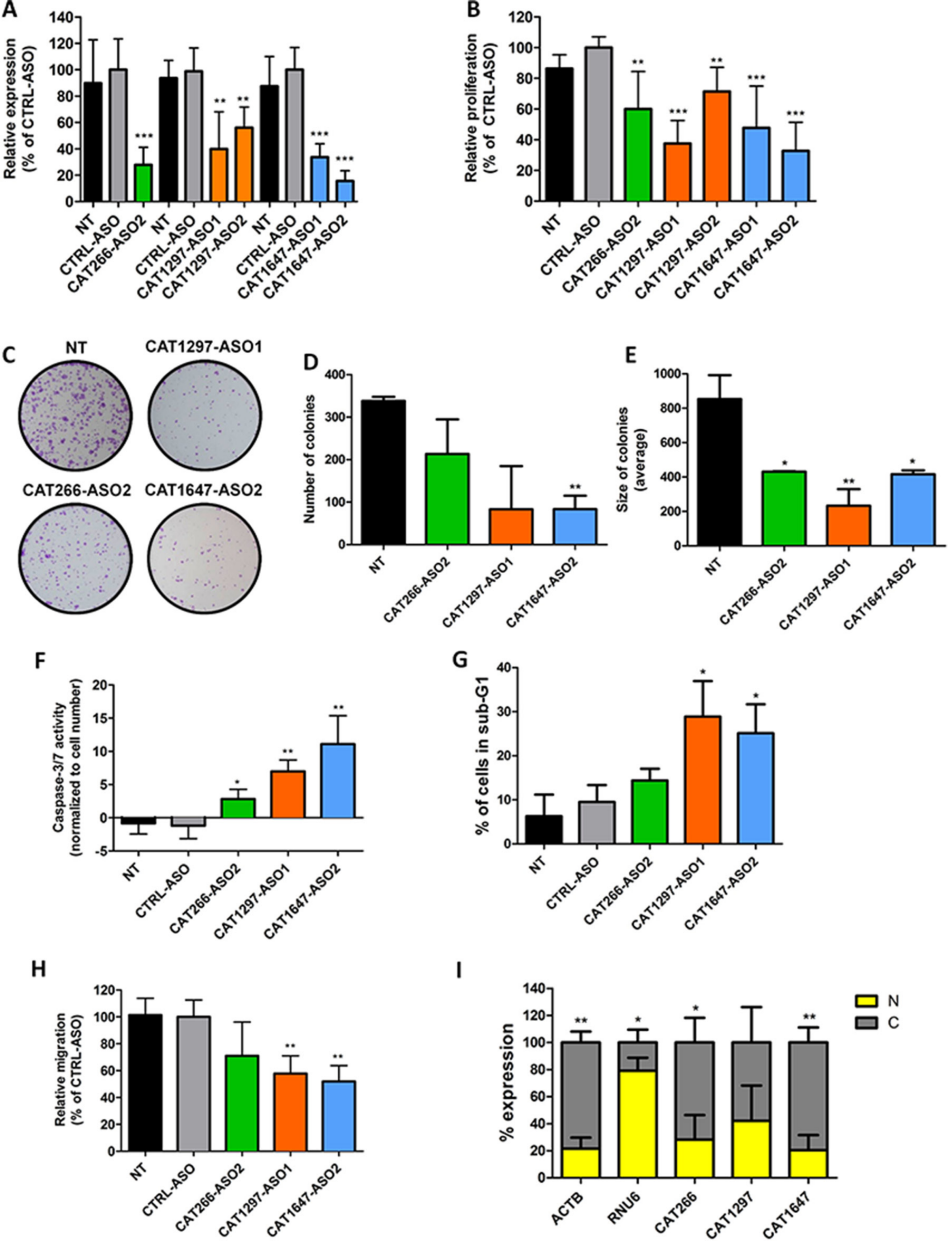
Knockdown of lncRNA expression (**A**) Expression levels of *CAT266*, *CAT1297* and *CAT1647* (determined by RT-qPCR) 48 hours after transfection of ASOs. The effect of *CAT266*, *CAT1297* and *CAT1647* ASO-mediated knockdown in SW780 cells on (**B**) cell proliferation, (**C**) colony formation, (**D**) number of colonies and (**E**) size of colonies. (**F**) Induction of apoptosis upon knockdown of *CAT266*, *CAT1297* and *CAT1647*, measured by an increase Caspase-3/7 activity. (**G**) Increase in sub-G1 cell population after knockdown of *CAT266*, *CAT1297* and *CAT1647*, analyzed by propidium iodide staining and flow cytometry. (**H**) The effect of *CAT266*, *CAT1297* and *CAT1647* ASO-mediated knockdown in SW780 cells on migration. All data are compared to control ASO-transfected cells. (t-test *p < 0.05; **p < 0.01; ***p < 0.001, bars represent the mean ± SD). (**I**) Expression of *CAT266*, *CAT1647* and *CAT1297* in nuclear (N) and cytoplasmic (C) RNA fractions. *ACTB* (cytoplasmic RNA) and *RNU6* (nuclear RNA) were used as controls for purity of isolated fractions (*t-test*, *p < 0.05; **p < 0.01, bars represent the mean ± SD).

To investigate the role of *CAT266*, *CAT1297* and *CAT1647*, we used antisense oligonucleotides (ASOs) and siRNAs to knockdown their expression. Upon ASO-mediated knockdown, significantly decreased expression levels of *CAT266*, *CAT1297* and *CAT1647* were found in comparison to control ASO-treated SW780 or SW800 cells (Figure 3A and Supplementary Figure 2, resp.). Reduced expression of *CAT266*, *CAT1297* and *CAT1647* significantly inhibited cell proliferation of SW780 (Figure 3B) and SW800 (Supplementary Figure 3) bladder cancer cells. SiRNA-mediated lncRNA knockdown in SW780 and SW800 bladder cancer cells was less efficient than ASO-mediated knockdown (Supplementary Figure 4). Consequently, the siRNA-mediated knockdown of *CAT266* resulted in the decreased cell viability, however to a much lower extent than observed when using ASOs (Supplementary Figure 5). Colony forming assay confirmed that knockdown of *CAT266*, *CAT1297* and *CAT1647* impaired growth of cancer cells (Figure 3C), as indicated by a reduction in number of colonies (Figure 3D) and a decreased size of the colonies (Figure 3E). The reduced cell viability was found to be a result of induction of apoptosis marked by an increase in Caspase-3/7 activity (Figure 3F) and an increase in the sub-G1 cell population (Figure 3G). Finally, cell migration capacity was also inhibited when *CAT1297* and *CAT1647* were knocked down in SW780 cells (Figure 3H).

### Cellular localization of *CAT266, CAT1297* and *CAT1647*

LncRNAs are known to be master regulators of gene expression. They may regulate transcript levels pre- and post-transcriptionally, depending on their cellular localization [6]. In SW780 bladder cancer cells, *CAT266* and *CAT1647* were found to be significantly enriched in the cytoplasmic fraction, whereas similar levels of *CAT1297* were found in both the cytoplasm and the nucleus (Figure 3I).

## DISCUSSION

More than 330,000 patients are diagnosed with bladder cancer worldwide every year. Almost half of the MIBC patients, despite receiving treatment, progress to metastatic disease and eventually die within 5 years [3]. The Cancer Genome Project revealed that gene mutations, copy number alterations and de-regulated expression of protein-coding genes and microRNAs occur frequently in MIBC [23]. In our study we focused on non-protein-coding genes and by analysis of the MiTranscriptome lncRNA expression data [5] we identified numerous, aberrantly expressed transcripts in bladder cancer tissue.

De-regulated expression of lncRNAs has been shown to be associated with all canonical hallmarks of cancer, including regulation of proliferation, apoptosis, invasion, metastasis and angiogenesis [24]. Two studies showed that expression levels of multiple, bladder cancer-specific lncRNAs correlated with expression of protein-coding genes involved in the p53 signalling pathway, mTOR signalling or in cell cycle regulation [18, 25]. Peter *et al*. identified 32 lncRNAs that are associated with bladder tumor progression. Remarkably, the number of deregulated transcripts increased with invasive phenotype and, in contrast to our study, the majority of transcripts (91%) were up-regulated. Knockdown of one of the identified lncRNA *AB074278* led to decreased cell proliferation and induction of apoptosis [26]. Similarly, knockdown of *CAT266*, *CAT1297* and *CAT1647* activated apoptotic pathways leading to decrease in migration capacity, which is an important step for cancer cells allowing them to invade and eventually form metastasis [27]. This indicates that muscle-invasive tumors are directly dependent on lncRNAs expression for sustaining growth, cell survival and cell invasion.

Furthermore, the importance of lncRNA expression in MIBC is supported by the correlation of *CAT1297* expression levels with progression-free and overall survival. Although up-regulation of *CAT1297* was shown to be associated with MIBC cell survival, high *CAT1297* levels correlated with longer overall survival. Currently, MIBC tumors are removed by cystectomy, and patients may receive cisplatin-based neo-adjuvant or adjuvant chemotherapy. The use of neo-adjuvant chemotherapy significantly increases OS [3]. As many lncRNAs have been shown to modify drug sensitivity [28, 29], it can be hypothesized that cells expressing *CAT1297* are more susceptible to chemotherapy. One of the limitations of our study is the lack of sufficient clinical data about treatment, therefore correlation of lncRNA expression with chemotherapy response could not be assessed. Several markers predicting chemotherapy response have been identified, including DNA repair gene alterations [30] or mRNA and protein gene signatures [31]. Discovery of lncRNAs predicting response to treatment and survival, will allow for patient stratification and improvement of MIBC clinical management.

NMIBC is characterized by high recurrence rate, leading to extensive follow-up of bladder cancer patients by cystoscopy. Urine is an ideal, non-invasive source for biomarker analysis, however, until now, no urinary marker is used in the clinic [2]. Two of the identified lncRNAs, *CAT266* and *CAT1297*, were found to be significantly up-regulated not only in MIBC but also in a subset of NMIBC. Furthermore, expression of *CAT266* and *CAT1297* was found to be cancer-specific as they were not expressed, or at low levels, in normal urothelium, normal prostate and normal kidney tissue. Therefore, the potential use of the two identified markers, and in addition other de-regulated lncRNAs that may be identified using the MiTranscriptome database, for non-invasive bladder cancer monitoring should be further evaluated in a larger set of NMIBC tissue samples and in urine collected from NMIBC patients.

Despite the overall low expression levels of lncRNAs in comparison to protein-coding mRNAs, they are able to regulate a variety of cellular processes [6]. *CAT266* is a novel lncRNA discovered by the MiTranscriptome study, and it located in a region in which no lncRNAs have been annotated before. *CAT1297* partially overlaps with a previously identified lncRNA, RP11-*190J1.3* and *CAT1647* with *RP11-1103G16.1*, but to our knowledge no data on *CAT266*, *CAT1297* and *CAT1647* function have been published so far. We have shown that *CAT266*, *CAT1297* and *CAT1647* are expressed in the cytoplasm and in the nucleus, suggesting that they modulate gene expression levels through different mechanisms. More and more data are becoming available about the role of lncRNAs in RNA-RNA and RNA-protein crosstalk. LncRNAs can act as competing endogenous RNAs (ceRNAs), by regulating other RNAs through binding of microRNAs [32, 33]. We analyzed potential lncRNA-microRNAs interactions using the online available DIANA [34] and NPinter tools [35]. Multiple microRNAs were predicted to bind to *CAT1297* and *CAT1647*, but these findings require further experimental validation in bladder cancer cell line models.

In conclusion, our data show that lncRNAs play an important role in bladder cancer development and progression, by regulating processes crucial for tumorigenesis, including cell proliferation and apoptosis. Further studies should focus on the identification of genes regulated by these bladder cancer-associated lncRNAs, in order to elucidate novel pathways involved in bladder tumorigenesis. A better understanding of the biology underlying MIBC may lead to the development of new treatment options for this life-threatening disease.

## MATERIALS AND METHODS

### Identification of MIBC-associated lncRNAs from the MiTranscriptome database

LncRNAs expression data (FPKM values) for muscle-invasive bladder cancer and normal urothelium tissue samples were downloaded from the MiTranscriptome database (http://mitranscriptome.org) [5]. Transcriptome data for the 16 normal urothelium and 122 MIBC tissue samples were generated in The Cancer Genome Atlas (TCGA) project. After exclusion of duplicates, expression data for 12,382 lncRNAs remained. Subsequently, 345 transcripts that were neither expressed in normal nor in cancer samples were excluded. From the remaining 12,037 lncRNAs, the most de-regulated transcripts were selected based on fold change in expression in normal and tumor samples (calculated using both average and median expression values) and based on *p*-values lower than 0.001 (unpaired *t-test*, IBM SPSS Statistics 22).

### Hierarchical clustering

Based on differences in expression levels between normal and MIBC tissue samples in the MiTranscriptome cohort (unpaired *t-test*, *p <* 0.1, unadjusted for multiple comparisons) 2,504 lncRNAs were selected for hierarchical clustering analysis. DNA mutation, copy number alteration and mRNA expression data for the patients and controls (that were included in the MiTranscriptome dataset) were downloaded from cBioPortal [36, 37]. Gene expression levels are highly skewed, and therefore, RNA expression FPKM values were log transformed and standardized by Z-score transformation per gene. These data were then visualized in a heat map that was generated using the heatmap.2 function from the gplots package in R (version 3.1.2).

### Cell culture

SW780 (ATCC# CRL-2169) and SW800 bladder cancer cell line [38] were grown in RPMI-1640 medium (Invitrogen) supplemented with 10% fetal calf serum (Sigma-Aldrich, F7524) and L-glutamine. Cell lines were cultured in a humidified atmosphere at 37°C and 5% CO_2_. SW780 and SW800 were tested for *Mycoplasma* and authenticated in 2016 using the PowerPlex 21 PCR kit (Promega) by Eurofins Genomics.

### Antisense oligonucleotide- and siRNA-mediated knockdown of lncRNA expression

Gapmer antisense oligonucleotides (ASOs) and small interfering RNAs (siRNAs) targeting up-regulated lncRNAs were designed using SFold software [39]. Twenty nucleotides long chimeric gapmers (RNA_5_-DNA_10_-RNA_5_) were chemically modified by addition of phosphorothioate linkages and introduction of 2’-O-Me groups in the flanking RNA parts (Eurogentec). Custom Silencer siRNAs were used (Thermo Fisher Scientific). Antisense oligonucleotide and siRNA sequences are listed in Supplementary Table 1. Bladder cancer cell lines were transfected at 70–80% confluency with 0.7 μM antisense oligonucleotide using X-tremeGENE 9 transfection reagent (Sigma-Aldrich) or with siRNAs at 20 nM final concentration using Lipofectamine RNAiMAX transfection reagent (Thermo Fisher Scientific), according to the manufacturer's instructions. Each experiment was performed at least three times. Non-transfected cells were treated with transfection reagent only. All experiments are compared to control ASOs or control siRNA (Silencer Negative Control No.1 siRNA) transfected cells.

### Tissue collection and processing

The use of patient materials was approved by the local ethics committee of the Radboud university medical center (CMO Arnhem-Nijmegen). Upon transurethral resection of tumor tissue (TUR) or cystectomy, specimens were snap frozen in liquid nitrogen. Normal urothelium (microdissected from bladder tissue obtained from bladder cancer patients after cystectomy, *n* = 5), non-muscle-invasive bladder (*n* = 18), and muscle-invasive bladder cancer (*n* = 47) specimens were selected for purity of benign or cancer cells, respectively, and processed by step sectioning. Patient and tumor characteristics can be found in Supplementary Table 2.

### RNA isolation and RT-qPCR

Total RNA was isolated using TRIzol reagent (Invitrogen) according to protocol. The RNA quantity and purity was evaluated using Nanodrop ND-1000 (Thermo Scientific). One microgram of RNA was DNaseI treated, and cDNA was synthesized using random hexamer primers and SuperScript II reverse transcriptase (Invitrogen). Gene expression levels were determined by SYBR Green qPCR (Roche) analysis in 16 μl reaction volumes, performed in duplicate, using a LightCycler LC480 instrument (Roche). RNA not subjected to reverse transcriptase was used as a negative control for PCR amplification. Human Heterochromatin Protein 1, Binding Protein 3 (*HP1BP3*) was used for normalization and relative gene expression levels were quantified using the ΔΔCt method. Primer sequences are listed in Supplementary Table 1.

### Cell viability assay

One day after transfection, cells were re-seeded into 96-well plates. Cell viability was assessed at regular time intervals using the CellTiter-Glo luminescence assay (Promega), according to the manufacturer's instructions. Luminescence was measured on the Victor^3^ multilabel reader (Perkin Elmer). Each experiment was performed in triplicate and repeated at least three times.

### Cell migration assay

Cell migration was determined using a “wound healing” scratch assay. One day post transfection, cells were seeded in 6-well plates at high cell density and cultured to 100% confluence within 24 hours. The next day, scratches were made using a sterile pipette tip. Cells were subsequently rinsed with 0.9% NaCl, standard culture media was added, and cells were allowed to migrate into the cell-free area. Microscopic images were taken at regular time intervals after applying the scratch. The mean closure of the scratch was calculated from three individual scratches per group at each time point. Each experiment was repeated at least three times.

### Colony formation assay

One day post transfection, cells were seeded in 6-well plates at a density of 500 cells/well, and cultured at standard conditions. After 12 days, colonies were washed with PBS, fixed with 3% paraformaldehyde and stained with 0.01% crystal violet (Merck) for 30 min. The number and size of colonies was analyzed using ImageJ software.

### Apoptosis assay

One day post transfection, cells were seeded into 96-well plates. The next day, Caspase-3/7 activity was measured using the Apo-ONE Homogenous Caspase-3/7 Assay (Promega), according to the manufacturer's instructions. After 2 h of incubation, luminescence was measured on a Victor^3^ multilabel reader (Perkin Elmer). The luminescence signals were normalized to cell number (assessed by CellTiter-Glo luminescence assay) and Caspase-3/7 activity of non-transfected cells was used as background. Each experiment was performed in triplicate and repeated at least three times.

### Cell-cycle analysis

One day post transfection, cells were seeded in 6-well plates. The next day cells were harvested, washed with 0.9% NaCl, resuspended in HBSS buffer (Invitrogen), and fixed with ice-cold ethanol. Fixed cells were centrifuged, resuspended in PBS and treated with RNase A (1 mg/ml, Sigma) for 40 min at 37^°^C. Subsequently, cells were stained with propidium iodide (400 μg/ml, Sigma) for 15 min in darkness. The samples were analyzed on a FC500 Flow Cytometer (Beckman-Coulter) and histograms were created using FlowJo software.

### Nuclear and cytoplasmic RNA isolation

Nuclear and cytoplasmic RNA was isolated using the Cytoplasmic and Nuclear RNA Purification Kit (Norgen Biotek), according to the manufacturer's instructions. The RNA quantity and purity was measured on a Nanodrop ND-1000 (Thermo-scientific). The integrity of the RNA fractions was evaluated by 1% agarose gel electrophoresis. RNA was used for cDNA synthesis and qPCR analysis as described above. Primers specific for *RNU6* (nuclear marker) and for *ACTB* (cytoplasmic marker) were used to assess the quality of the RNA fractions (primer sequences are listed in Supplementary Table 1). Experiments were repeated three times.

### Statistical analysis

All statistical analyses was performed using GraphPad Prism or IBM SPSS software, and *p <* 0.05 was considered statistically significant. For lncRNA expression data, an unpaired *t-test* was used. In knockdown experiments, data were compared to control ASO-transfected cells or control siRNA-transfected cells and unpaired *t-test* was performed. Kaplan-Meier curves were analyzed using a Log-Rank (Mantel-Cox) test and Cox proportional hazard regression analyses were performed. Clinical data for bladder cancer patients included in the MiTranscriptome database were downloaded from cBioPortal [36, 37].

## SUPPLEMENTARY MATERIALS FIGURES AND TABLES


