# Supplementary material for: Identification of long non-coding RNAs that stimulate cell survival in bladder cancer
Source: Oncotarget. 2017 Mar 16;8(21):34442–52. doi: 10.18632/oncotarget.16284 (PMC5470981; doi:10.18632/oncotarget.16284)
Supplement: Supplementary file 1 [file oncotarget-08-34442-s001.pdf]

Identification of long non-coding RNAs that stimulate cell survival in bladder cancer

Supplementary Materials

Supplementary Table 1: Primers, antisense oligonucleotide (ASO) and siRNAs sequences

**Primers**		
**Gene**	**Forward primer (5′→3′)**	**Reverse primer (5′→3′)**
**ACTB**	CGTCTTCCCCTCCATCGTG	GATGGGGTACTTCAGGGTGA
**CAT266**	GCTCATTGCCCTCAAGTGTT	AGGACTGCCTGAGACCATGA
**CAT1297**	TGGGATTACAAAATGCGTGA	CATGGCAGCAGCAAGTAGAA
**CAT1647**	GGCTAACAGATGCCCAGATG	AACTGGCCACAGCAGAGATT
**HP1BP3**	TGGAATATGCAATCTTGTCTGC	GAACCCTTTCCCAGAGATCTG
**RNU6**	CGCTTCGGCAGCACATATAC	AGGGGCCATGCTAATCTTCT
**Antisense oligonucleotides**		
**Name**	**Target sequence (5′→3′)**	**Antisense oligo (5′→3′)**
**CAT266-ASO1**	CAACAAUGGAGCCCCAAGUG	CACTTGGGGCTCCATTGTTG
**CAT266-ASO2**	ACAUUGGCAUUAGCCUUAUC	GATAAGGCTAATGCCAATGT
**CAT1297-ASO1**	GAAAACAAUUCACCUCUUGC	GCAAGAGGTGAATTGTTTTC
**CAT1297-ASO2**	GUUCUCUACAACCUCCACCU	AGGTGGAGGTTGTAGAGAAC
**CAT1647-ASO1**	UCACUGUCUCUUACUAAAGC	GCTTTAGTAAGAGACAGTGA
**CAT1647-ASO2**	GAUUCAAUUGCUCACCUCUU	AAGAGGTGAGCAATTGAATC
**CTRL-ASO1 (38)**	*none*	AAGCGCGCACCAGCGCCTCC
**CTRL-ASO2 (39)**	*none*	CCTTCCCTGAAGGTTCCTCC
**siRNAs**		
**Name**	**Sense siRNA (5′→3′)**	**Antisense siRNA (5′→3′)**
**CAT266-siRNA**	GCUGGAAUUACAGGUAUAATT	UUAUACCUGUAAUUCCAGCTT
**CAT1297-siRNA1**	CUCAUGACAACUCUUAUUUTT	AAAUAAGAGUUGUCAUGAGTT
**CAT1297-siRNA2**	GAAACACACACACACUCUUTT	AAGAGUGUGUGUGUGUUUCTT
**CAT1647-siRNA1**	ACUGUCUCUUACUAAAGCUTT	AGCUUUAGUAAGAGACAGUTT
**CAT1647-siRNA2**	GGAAACACCUUCACAGACUTT	AGUCUGUGAAGGUGUUUCCTT
**CTRL-siRNA 1**	Silencer Negative Control No.1	(AM4611, Ambion)

Supplementary Table 2: Clinical characteristics of patients used for validation

	NMIBC n = 18		MIBC n = 47
**Age** (average, range)	61 (50–77)	**Age** (average, range)	67 (37–84)
**Gender**		**Gender**	
female	4	female	15
male	14	male	32
**Stage**		**Stage**	
pTa	9	pT2	11
pT1	7	pT3	22
CIS	2	pT4	12
		unknown	2
**Grade**		**Grade**	
G1	1	G2	5
G2	9	G3	36
G3	6	unknown	6
unknown	2		

Supplementary Table 3: The 25 most up-regulated lncRNAs in MIBC identified based on average expression levels using MiTranscriptome data

Transcript[#]	Name	Average expression		T/N ratio	Median expression		T/N ratio	*P*-value[$]
		Benign (N)	Cancer (T)		Benign (N)	Cancer (T)		
T102457	***CAT1647.6***	0	0.155	> 100	0	0.069	> 100	< 0.001
T047629	***CAT1297.4***	0	0.128	> 100	0	0.077	> 100	< 0.001
T078005	***CAT1507***	0.001	0.296	> 100	0	0.096	> 100	< 0.001
T290558	***THCAT68.1***	0.002	0.128	60.98	0	0.042	> 100	< 0.001
T253942	***CAT467.2***	0.002	0.132	53.84	0	0.053	> 100	< 0.001
T235647	***CAT2260.3***	0.003	0.108	38.80	0	0.045	> 100	< 0.001
T108430	***CAT1695***	0.005	0.138	29.88	0	0.020	> 100	< 0.001
T047638	***LACAT97***	0.030	0.777	26.03	0	0.474	> 100	< 0.001
T065634	***CAT1409***	0.005	0.139	25.92	0	0.068	> 100	< 0.001
T207213	***CAT367***	0.035	0.887	25.31	0.011	0.367	31.71	< 0.001
T247881	***LSCAT103***	0.007	0.174	24.44	0	0.029	> 100	< 0.001
T318530	***CAT904.4***	0.006	0.147	24.44	0	0.075	> 100	< 0.001
T087060	***CAT1573***	0.006	0.137	23.60	0	0.024	> 100	< 0.001
T362906	***CAT1167***	0.005	0.114	23.50	0	0.019	> 100	< 0.001
T256721	***CAT491.2***	0.005	0.104	22.13	0	0.056	> 100	< 0.001
T074211	***LSCAT131***	0.005	0.114	21.28	0	0.055	> 100	< 0.001
T108891	***BRCAT116***	0.008	0.166	19.50	0	0.048	> 100	< 0.001
T196786	***MIR4435–1HG.1***	0.008	0.159	19.47	0	0.032	> 100	< 0.001
T078121	***LSCAT167***	0.013	0.242	18.70	0	0.142	> 100	< 0.001
T332213	***LSCAT55***	0.007	0.126	18.69	0	0.049	> 100	< 0.001
T100203	***STCAT7***	0.059	1.075	18.11	0	0.349	> 100	< 0.001
T220458	***CAT2182***	0.012	0.212	18.06	0.002	0.145	65.69	< 0.001
T332495	***CAT971***	0.016	0.281	17.56	0.008	0.129	16.21	< 0.001
T286785	***LINC00491.3***	0.008	0.132	17.34	0	0.063	> 100	< 0.001
T255259	***CAT475.3***	0.009	0.148	17.08	0	0.054	> 100	< 0.001
T192655	***CAT266.3****	0.26	1.84	6.95	0	1.49	> 100	< 0.001

[#]MiTranscriptome identifier; [$]unpaired *t*-test; [*]selected based on the highest T/N ratio calculated using median expression. The lncRNAs with average expression in cancer higher than 0.1 FPKM are shown.

Supplementary Table 4: Expression levels of 11 bladder cancer-associated lncRNAs that are described in literature, and for which expression data are available in the MiTranscriptome dataset

Transcript	Ref	Average expression*		T/N ratio	Median expression*		T/N ratio	*P*-value$
		Benign (N)	Cancer (T)		Benign (N)	Cancer (T)		
ANRIL	(17)	0.01	0.166	14.27	0.005	0.04	7.02	< 0.001
SChLAP1	(18)	0.06	0.31	5.1	0	0	1	0.014
HIF1A-AS2	(19)	0.02	0.07	3.5	0.02	0.02	–1.20	0.001
BC072678	(20)	0.05	0.18	3.49	0.04	0.06	1.58	< 0.001
PCAT1	(21)	0.01	0.04	2.94	0.01	0.02	1.97	< 0.001
TINCR	(22)	2.06	5.59	2.71	0.59	3.42	5.72	< 0.001
MALAT1.2	(23)	4.63	12.16	2.62	0.08	5.81	78.32	0.033
HOTAIR	(40)	0.005	0.009	1.94	0	0	1	0.133
TUG1.4	(24)	11.99	22.93	1.91	8.89	18.34	2.06	< 0.001
ZEB2NAT	(41)	0.05	0.02	-3,16	0.05	0	> 100	0.001
HOXD-AS1	(42)	1.49	1.18	-1.26	0.25	0.52	–2.1	0.631
NEAT1	(43)	11.22	8.76	-1.28	4.75	2.16	2.19	0.585

*MiTranscriptome expression data; $unpaired *t*-test.

Supplementary Table 5: Correlation of clinico-pathological parameters with expression levels of the three identified MIBC-associated lncRNAs

Parameter	No*	*CAT266*		*CAT1297*		*CAT1647*	
		Average expression	*P*-value$	Average expression	*P*-value$	Average expression	*P*-value$
Age (years)							
**< 60**	31	1.601	0.407	0.111	0.426	0.115	0.210
**> 60**	91	1.922		0.134		0.164	
Sex							
**Female**	34	2.169	0.259	0.079	0.008	0.125	0.311
**Male**	88	1.713		0.147		0.166	
Lymph nodes							
**Positive (N1–3)**	40	2.049	0.412	0.130	0.940	0.176	0.745
**Negative (N0)**	71	1.722		0.129		0.155	
Stage							
**pT2**	30	1.979	0.746	0.147	0.718	0.288	**0.015**
**pT3**	62	1.938		0.117		0.117	
**pT4**	16	1.529		0.123		0.117	
Grade							
**low**	6	1.184	0.411	0.110	0.867	0.049	**0.027**
**high**	115	1.863		0,120		0.144	
Subtype							
**Papillary**	33	1.916	0.882	0.166	0.226	0.208	0.354
**Non-papillary**	85	1,856		0.115		0.138	
Smoking							
**No smokers**	28	1.717	0.308	0.097	0.127	0.091	0.147
**Current/former smokers**	88	2.156		0.139		0.175	

*not all data available for all patients; $unpaired *t*-test.

Supplementary Table 6: Univariate and multivariate analysis of prognostic factors in MIBC for overall survival

Prognostic factor	Univariate analysis			Multivariate analysis		
	Exp (B)	95% CI	*P* value	Exp (B)	95% CI	*P* value
**CAT1297 expression**	0.487	0.292–0.812	0.006	0.508	0.284–0.909	0.022
**Age**	1.027	1.002–1.053	0.036	1.034	1.004–1.064	0.026
**Gender**	0.967	0.553–1.691	0.906	0.719	0.374–1.380	0.321
**Stage**	1.952	1.242–3.067	0.004	1.975	1.188–3.285	0.009
**Lymph node metastasis***	1.725	1.020–2.919	0.042	1.434	0.374–2.478	0.197

*At the time of diagnosis, CI-confidence intervals.

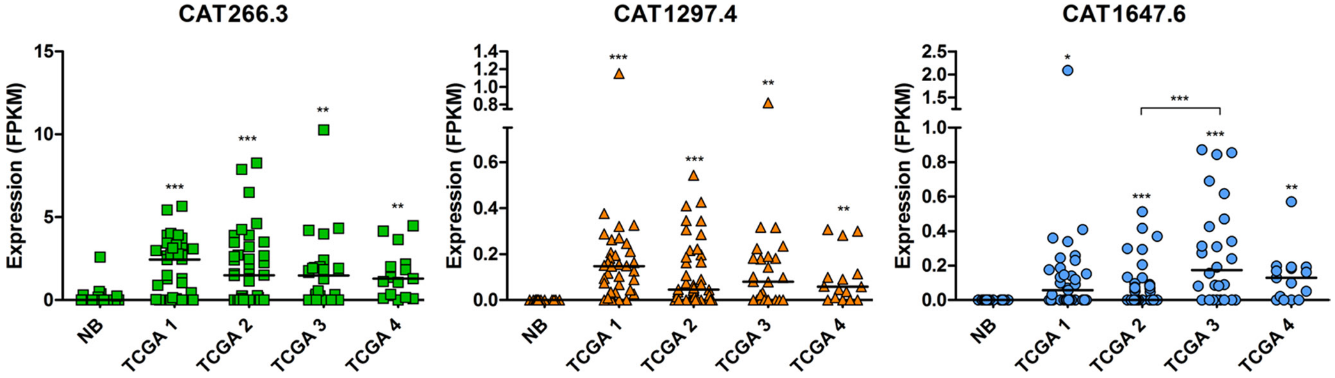

Supplementary Figure 1: Expression of *CAT266, CAT1297* and *CAT1647* in normal bladder (NB) and MIBC tumors divided into TCGA clusters (TCGA 1-4; unpaired *t* -test, **p* < 0.05; ***p* < 0.01, ****p* < 0.001; lines represent the median).

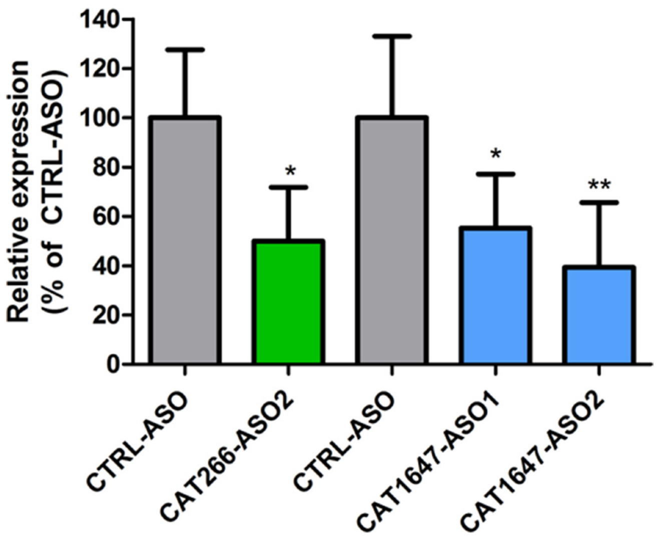

Supplementary Figure 2: Knockdown of lncRNAs expression in SW800 bladder cancer cell line (expressing *CAT266* and *CAT1647*) using ASOs. All data are compared to control ASO-transfected cells. (*t*-test, **p* < 0.05, ****p* < 0.001, bars represent the mean ± SD).

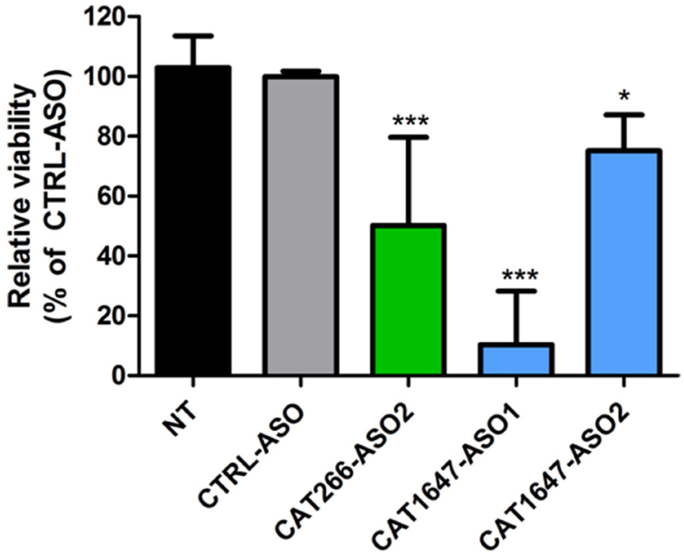

Supplementary Figure 3: The effect of *CAT266* and *CAT1647* ASO-mediated knockdown in SW800 cells on cell viability. All data are compared to control ASO-transfected cells. (*t*-test, *$p < 0.05$, ***$p < 0.001$, bars represent the mean ± SD).

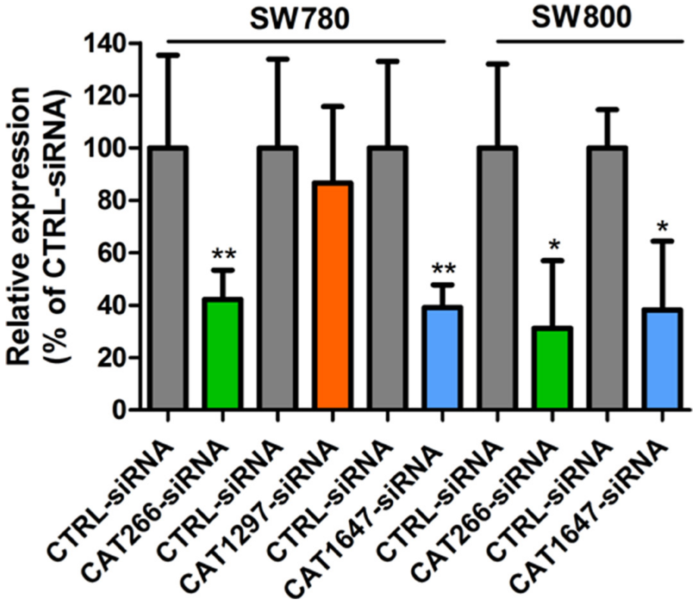

Supplementary Figure 4: Knockdown of lncRNAs expression using siRNAs targeting *CAT266* (CAT266-siRNA), *CAT1297* (CAT1297-siRNA1 and CAT1297-siRNA2 mix) and CAT1647 (CAT1647-siRNA1 and CAT1647-siRNA2 mix) in SW780 and SW800 bladder cancer cell lines. All data are compared to control siRNA-transfected cells (*t*-test, *$p < 0.05$; **$p < 0.01$, bars represent the mean ± SD).

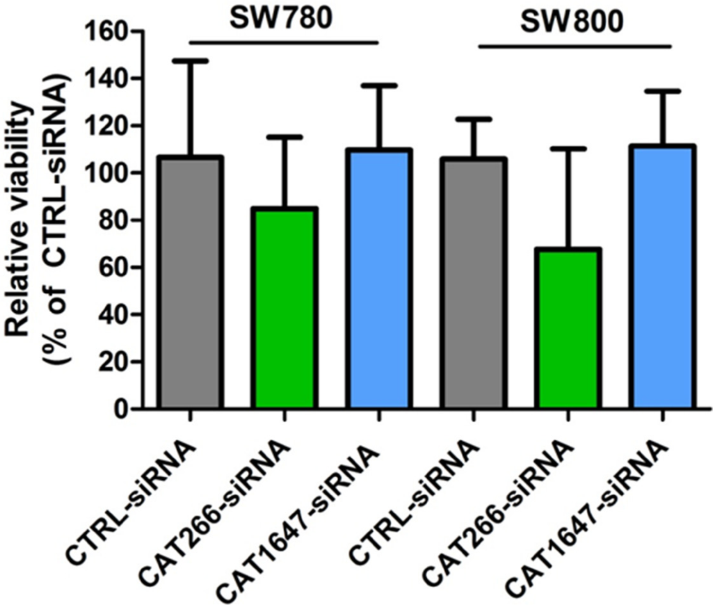

Supplementary Figure 5: The effect of *CAT266* (CAT266-siRNA1) and *CAT1647* (CAT1647-siRNA1 and CAT1647-siRNA2 mix) siRNA-mediated knockdown on cell viability in SW780 and SW800 bladder cancer cell lines. All data are compared to control siRNA-transfected cells (*t* -test, bars represent the mean ± SD).
